# The Promoter of the Immune-Modulating Gene *TIR-Containing Protein C* of the Uropathogenic *Escherichia coli* Strain CFT073 Reacts to the Pathogen’s Environment

**DOI:** 10.3390/ijms23031148

**Published:** 2022-01-20

**Authors:** Jacqueline Hemberger, Julia Ittensohn, Hannah Griffiths, Maren Keller, Victor Costina, Simone Albrecht, Thomas Miethke

**Affiliations:** 1 Medical Faculty of Mannheim, Institute of Medical Microbiology and Hygiene, University of Heidelberg, Theodor-Kutzer-Ufer 1-3, 68167 Mannheim, Germany; Jacqueline.Hemberger@medma.uni-heidelberg.de (J.H.); julia.ittensohn@umm.de (J.I.); Hannah.Griffiths@medma.uni-heidelberg.de (H.G.); Maren.Keller@medma.uni-heidelberg.de (M.K.); Simone.Albrecht@medma.uni-heidelberg.de (S.A.); 2 Medical Faculty of Mannheim, Mannheim Institute for Innate Immunoscience (MI3), University of Heidelberg, Ludolf-Krehl-Str. 13–17, 68167 Mannheim, Germany; victor.costina@medma.uni-heidelberg.de; 3 Medical Faculty of Mannheim, Institute of Clinical Chemistry, University of Heidelberg, Theodor-Kutzer-Ufer 1-3, 68167 Mannheim, Germany

**Keywords:** uropathogenic *Escherichia coli*, TcpC, host cell, potassium, sodium, bacterial density

## Abstract

The *TIR-containing protein C* (TcpC) of the uropathogenic *Escherichia coli* strain CFT073 modulates innate immunity by interfering with the Toll-like receptor and NALP3 inflammasome signaling cascade. During a urinary tract infection the pathogen encounters epithelial and innate immune cells and replicates by several orders of magnitude. We therefore analyzed whether these cell types and also the density of the pathogen would induce the recently defined promoter of the CFT073 *tcpC* gene to, in time, dampen innate immune responses. Using reporter constructs we found that the uroepithelial cell line T24/83 and the monocytic cell line THP-1 induced the *tcpC* promoter. Differentiation of monocytic THP-1 cells to macrophages increased their potential to switch on the promoter. Cell-associated CFT073 displayed the highest promoter activity. Since potassium represents the most abundant intracellular ion and is secreted to induce the NLRP3 inflammasome, we tested its ability to activate the *tcpC* promoter. Potassium induced the promoter with high efficiency. Sodium, which is enriched in the renal cortex generating an antibacterial hypersalinity, also induced the *tcpC* promoter. Finally, the bacterial density modulated the *tcpC* promoter activity. In the search for promoter-regulating proteins, we found that the DNA-binding protein H-NS dampens the promoter activity. Taken together, different cell types and salts, present in the kidney, are able to induce the *tcpC* promoter and might explain the mechanism of TcpC induction during a kidney infection with uropathogenic *E. coli* strains.

## 1. Introduction

The *Escherichia coli* strain CFT073 was isolated from a patient with acute pyelonephritis [1]. The completely sequenced genome of this strain contains a bacterial *TIR-containing protein* (Tcp) termed TcpC which binds to Toll-like receptor 4 (TLR4) and myeloid differentiation factor 88 (MyD88) as well as NACHT leucin-rich repeat PYD protein 3 (NLRP3) and caspase-1 [2,3,4,5]. Moreover, TcpC serves as an E3 ubiquitin ligase, promotes ubiquitination of MyD88 and its co-localization with the proteasome, and lowers intracellular MyD88 levels in kidney macrophages [6]. A number of bacterial pathogens contain Tcps in their genomes which share the ability of TcpC to interfere with the TLR signaling cascade and to act as virulence factors [5,7,8,9].

We reported earlier that CFT073 causes kidney abscesses TcpC-dependently in mice that contain an accumulation of neutrophils and bacteria [5,10]. We and others also found that uropathogenic *E. coli* (UPEC) strains isolated from patients with pyelonephritis contain the *tcpC* gene in around 40% of cases but in only 21 and 16% in cases of cystitis or asymptomatic bacteriuria, respectively [5,11]. Thus, the number of bacterial isolates harboring the *tcpC* gene correlates with the disease severity of the urinary tract.

We recently defined the promoter regions controlling the expression of the *tcpC* gene. We used green fluorescence protein (*gfpmut2*)-reporter constructs which contain a 645 bp element starting at position 2200564 and ending at position 2201209 of the CFT073 genome, designated promoter 1 (P1), and a 240 bp element starting at position 2202535 and ending at position 2202775 of the chromosome of CFT073, designated P2. The former is located in the 5′ UTR of the gene *c2397*, the latter in the 5′ UTR of *c2398*, which encodes TcpC. We found that P2 responded much more strongly than P1 to stimuli such as high pH, glucose concentration of 3 mmol/l or more, and urine [12]. These stimuli not only activated the plasmid-based reporter constructs transformed into CFT073 on plasmids but also stimulated a chromosomal reporter strain where we replaced *tcpC* by *gfpmut2*. We mapped the transcription start of P2 44 bp 5′ of a putative start codon of the *c2398* RNA [12]. 

Human kidneys consist of around one million nephrons which are composed of different epithelial cells. The organ also harbors a dense network of resident macrophages, which represent a major cellular component of the local innate immune system. The kidney medulla reabsorbs water and sodium and generates a hypersalinity, which in turn enhances the antibacterial function of monocyte-derived mononuclear phagocytes [13]. Epithelial cells secrete chemokines in hypersaline conditions and, by that, attract monocyte-derived mononuclear phagocytes [13]. Taken together, the high salt gradient is able to prevent organ infection. It thus appears that a UPEC strain infecting the kidneys is confronted with at least three basic hindrances: (i) the epithelial barrier of the collecting ducts and the tubular system of the nephrons, (ii) resident macrophages, and (iii) the antibacterial hypersalinity, which appears to drive the local defense system. Since TcpC seems to promote kidney infections by UPECs, we explored, whether the human uroepithelial cell line T24/83 and the human monocytic cell line THP-1 as well as sodium and potassium chloride are able to stimulate the *tcpC* promoter. The burden of the bacterial pathogens increases significantly during a urinary tract infection. Therefore, we also analyzed the role of the bacterial density in stimulating the *tcpC* promoter. We focused our analysis on P2 since it responded much more vigorously than P1. 

## 2. Results

CFT073 senses the human uroepithelial cell T24/83 and the human monocytic cell line THP-1.

**Figure 1:**
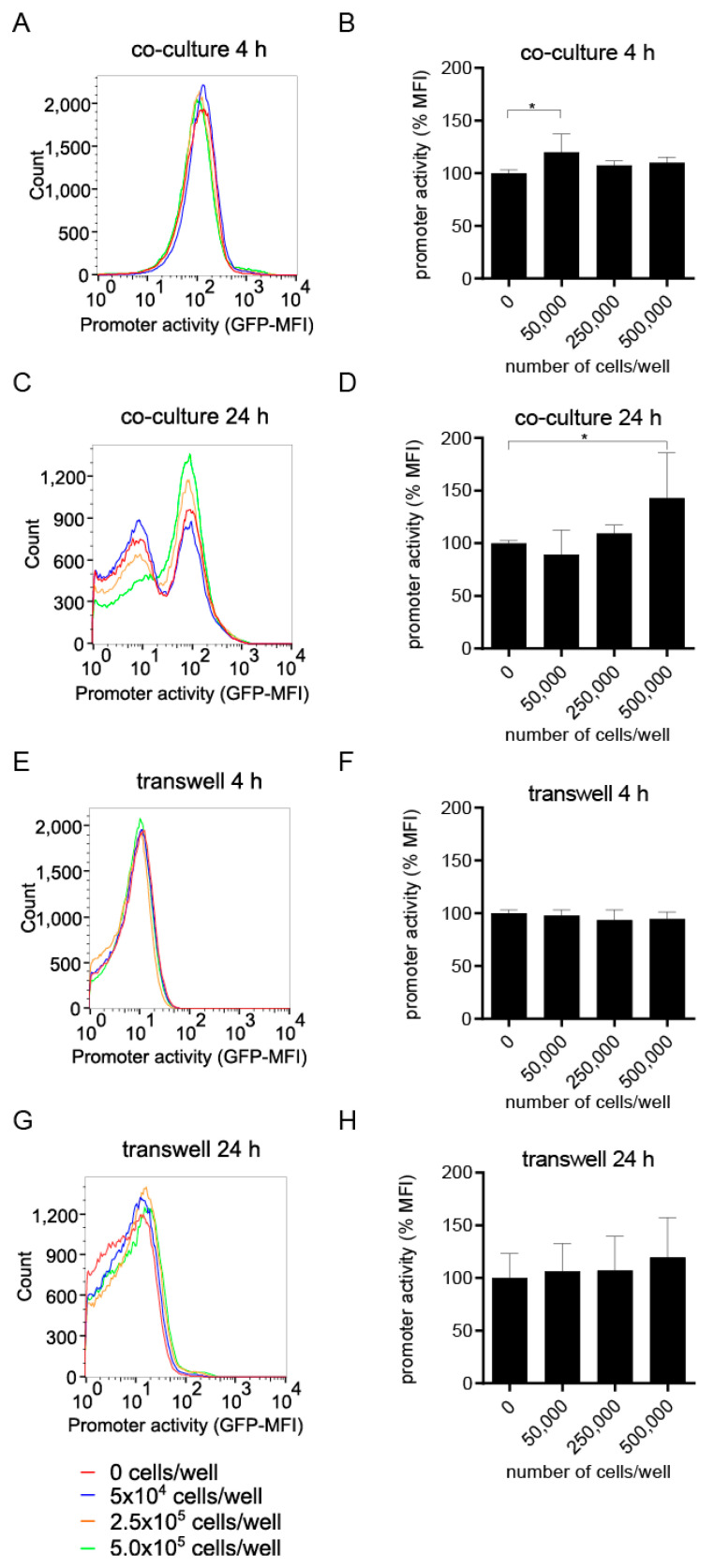
The epithelial cell line T24/83 induces the P2 promoter. We cultured CFT073 pPc2398:gfpmut:KAN in McCoy medium in the absence or presence of titrated amounts of T24/83 cells as indicated. Bacteria and T24/83 cells were either co-cultured (1.6 × 10^7^ bacteria/well, (**A**–**D**)) or cultured in transwells (1.6 × 10^8^ bacteria/well, (**E**–**H**)). We determined the P2 promoter activity after a culture period of 4 (**A**,**B**,**E**,**F**) or 24 h (**C**,**D**,**G**,**H**) by flow cytometry. (**A**,**C**,**E**,**G**) depict representative flow cytometry results. (**B**,**D**) show three independent experiments and (**F**,**H**) four independent experiments, each experiment was performed with three replicates. The measured promoter activity in (**B**,**D**,**F**,**H**) was normalized to the “0” control. * *p* < 0.05, ANOVA post-hoc Tukey.

TcpC impairs innate immune cells by binding to key components of the TLR-signaling cascade and the NLRP3 inflammasome [3,4,7]. We hypothesized that the virulence factor should be produced before innate immune cells attack the bacterial pathogen CFT073. Thus, epithelial cells of the urinary tract, which attract innate immune cells such as macrophages, potentially induce the virulence factor of CFT073. We therefore tested the ability of the uroepithelial cell line T24/83 to activate the *tcpC* promoter. We transformed CFT073 with the reporter plasmid Pc2398:gfpmut2 containing the P2 promoter fused to the green fluorescent protein mut2 (*gfpmut2*), described above, to generate the plasmid-based reporter strain CFT073 pPc2398:gfpmut2:KAN [12]. We then co-incubated the CFT073 pPc2398:gfpmut2:KAN reporter strain for 4 and 24 h with titrated amounts of T24/83 cells in McCoy medium and measured its fluorescence intensity by flow cytometry. We found that T24/83 cells influenced P2 after 4 h of co-incubation only to a minor extent (Figure 1A,B). Upon 24 h of culture of the CFT073 pPc2398:gfpmut2:KAN reporter strain in the absence of T24/83 cells, P2 activity was substantially lower (Figure 1C) compared to its activity at 4 h (Figure 1A). In this case T24/83 cells induced P2 dose-dependently (Figure 1C) and significantly (Figure 1D). We repeated the experiment using transwell plates, seeding bacteria in the top, and havingT24/83 cells in the lower chamber. The reporter was not or only slightly induced without statistical significance after 4 (Figure 1E,F) or 24 h (Figure 1G,H) of culture, respectively. This indicated that a contact of CFT073 and the epithelial cell line was required for P2 induction. 

**Figure 2:**
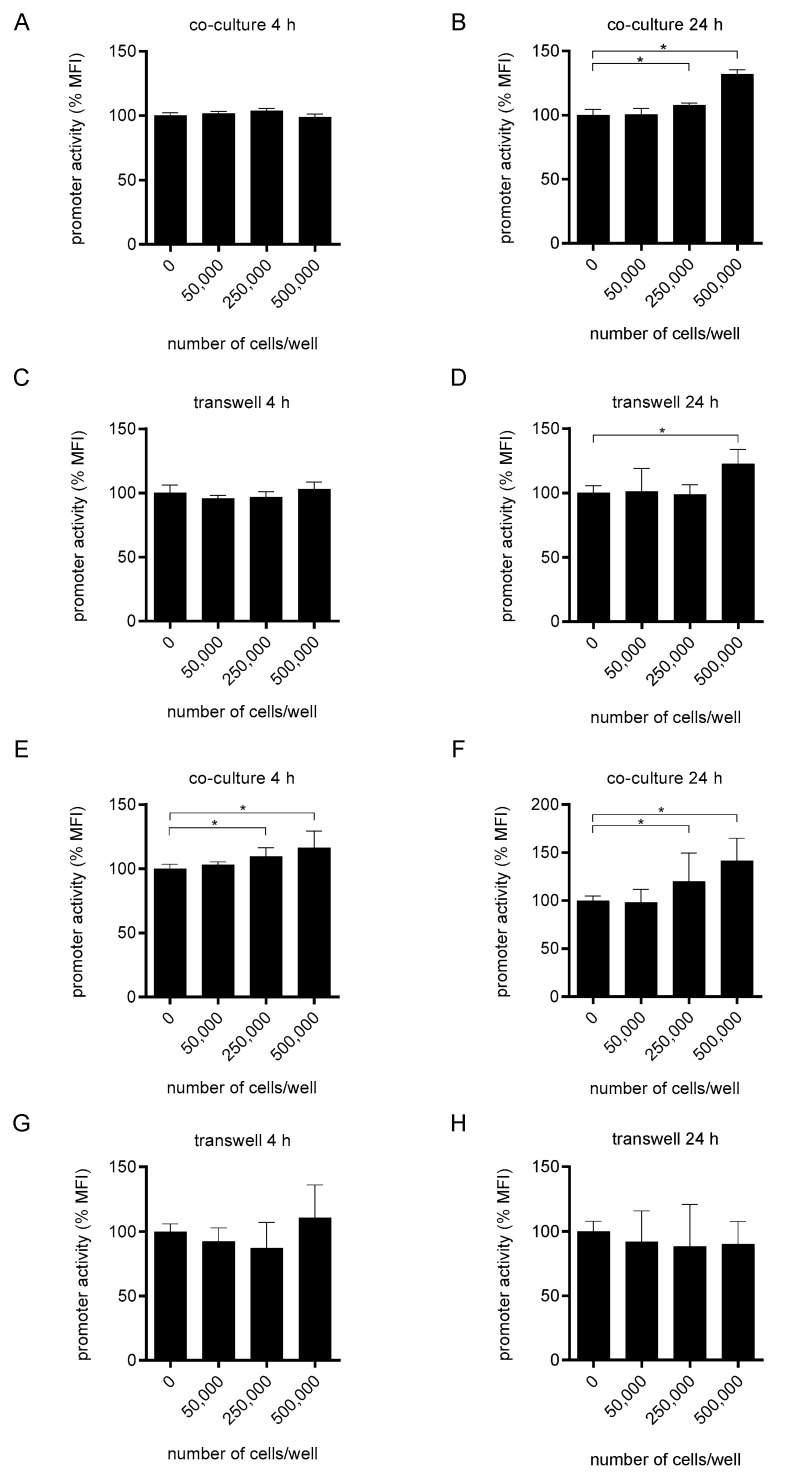
Differentiation of the monocytic cell line THP-1 to macrophages increases their efficacy to induce the P2 promoter. We cultured CFT073 pPc2398:gfpmut:KAN in RPMI-1640 medium in the absence or presence of titrated amounts of LPS-stimulated (100 ng/mL) monocytic THP-1 cells as indicated. Bacteria and THP-1 cells were either co-cultured (1.6 × 10^7^ bacteria/well, (**A**,**B**)) or cultured in transwells (1.6 × 10^8^ bacteria/well, (**C**,**D**)). We determined the P2 promoter activity after a culture period of 4 (**A**,**C**) or 24 h (**B**,**D**) by flow cytometry. In (**E**–**H**) we repeated the experiments described in (**A**–**D**) with the exception that we used THP-1 cells, which we differentiated with PMA (100 ng/mL) for three days. Cells were not stimulated with LPS. Graphs depict three (**A**–**D**,**G**,**H**) or six (**E**,**F**) independent experiments, each experiment was performed with three replicates. The measured promoter activity in (**A**–**H**) was normalized to the “0” control. * *p* < 0.05, ANOVA post-hoc Tukey.

We then analyzed the ability of the human monocytic cell line THP-1, which we stimulated with LPS, to induce the *tcpC* promoter. Co-cultivation of the CFT073 pPc2398:gfpmut2:KAN reporter strain with titrated amounts of THP-1 cells in RPMI 1640 (Figure 2A,B) but also incubation in transwells (Figure 2C,D) increased the promoter activity modestly but significantly after 24 but not 4 h of culture (Figure 2). We then tested whether maturation of THP-1 cells to macrophages by PMA treatment would change the influence of these cells on the *tcpC* promoter activity. We found that matured THP-1 cells increased the promoter activity dose-dependently and significantly after 4 h of co-culture (Figure 2E) and also after 24 h of co-culture (Figure 2F). Flow cytometry further revealed that the majority of the bacteria augmented P2 activity (Figure S1). This interaction required contact between the macrophages and the pathogen (Figure 2G,H). 

**Figure 3:**
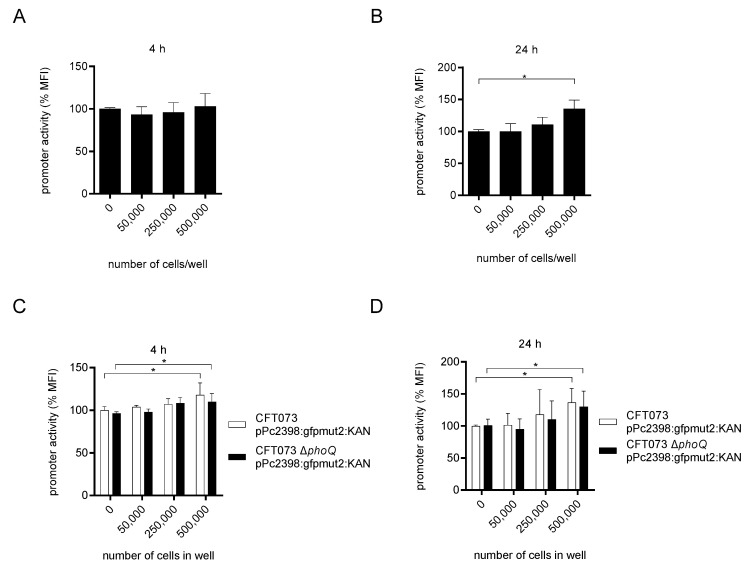
The PhoQ/P two-component system does not influence P2 induction by THP-1 cells. We co-cultured CFT073 Δ*phoQ* pPc2398:gfpmut:KAN (1.6 × 10^7^ bacteria/well) in RPMI-1640 medium in the absence or presence of titrated amounts of monocytic THP-1 cells as indicated (**A**,**B**). We determined the P2 promoter activity after a culture period of 4 (**A**) or 24 h (**B**) by flow cytometry. In (**C**,**D**) we differentiated THP-1 cells with PMA (100 ng/mL) for three days. Subsequently, we co-cultured wild-type CFT073 pPc2398:gfpmut:KAN (1.6 × 10^7^ bacteria/well) or CFT073 Δ*phoQ* pPc2398:gfpmut:KAN (1.6 × 10^7^ bacteria/ well) in the absence or presence of titrated amounts of differentiated THP-1 cells for 4 (**C**) or 24 h (**D**) as indicated and determined P2 promoter activity by flow cytometry. Graphs depict three independent experiments. Each experiment was performed with three replicates. The measured promoter activity in (**A**–**D**) was normalized to the “0” control. * *p* < 0.05, ANOVA post-hoc Tukey.

The two-component system (TCS) PhoQ/P senses macrophages and antibacterial peptides in *Salmonella* [14,15,16] and reduces the expression of a variety of genes via the regulatory non-coding small RNA MgrR in *E. coli* [17]. We therefore tested its involvement in the regulation of P2 during direct contact with THP-1 and matured THP-1 cells by generating the *phoQ*-deficient CFT073 Δ*phoQ* mutant (Figure S2A). This mutant was transformed with the reporter plasmid pPc2398:gfpmut2:KAN. We found that CFT073 Δ*phoQ* pPc2398:gfpmut2:KAN upregulated P2 upon co-culture with THP-1 cells for 24 but not 4 h (Figure 3A,B). Moreover, matured THP-1 cells induced the P2 promoter activity in CFT073 Δ*phoQ* pPc2398:gfpmut2:KAN similar to CFT073 pPc2398:gfpmut2:KAN (Figure 3C,D). Thus, the PhoQ/P TCS is apparently not involved in the regulation of the P2 promoter upon stimulation with THP-1 cells.

### 2.1. THP-1 Cell-Associated CFT073 Activate P2 Most Efficiently

**Figure 4:**
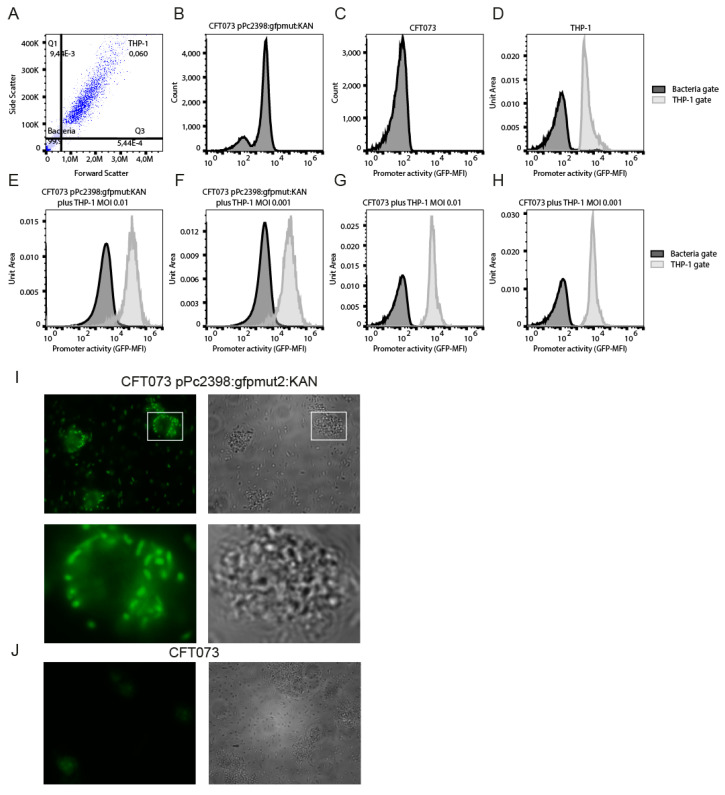
CFT073 in close association with THP-1 cells activate P2 most intensively. We co-cultured CFT073 pPc2398:gfpmut:KAN and monocytic THP-1 cells in RPMI-1640 medium and defined by forward and side scatter the bacterial ((**A**), lower left quadrant: Bacteria) and cellular gate ((**A**), upper right quadrant: THP-1). (**B**) displays just CFT073 pPc2398:gfpmut:KAN (5 × 10^3^ bacteria/well), (**C**) just CFT073 (5 × 10^3^ bacteria/well) and (**D**) just monocytic THP-1 cells (5 × 10^5^ cells/well), which were used as controls. In parallel we co-cultured monocytic THP-1 cells (5 × 10^5^ cells/well) with CFT073 pPc2398:gfpmut:KAN (MOI = 0.01 or 0.001, (**A**,**E**,**F**)) or with CFT073 (MOI = 0.01 or 0.001, (**G**,**H**)) as indicated. We determined P2 promoter activity by flow cytometry after 24 h. Using the bacteria and THP-1 gate as defined in (**A**) we determined THP-1-non-associated (dark gray) and THP-1-associated bacteria (light gray). The dark gray area in (**D**) represents cellular debris of THP-1 cells. We modified the experiment described above and co-cultured monocytic THP-1 cells (5 × 10^5^ cells/well) with CFT073 pPc2398:gfpmut:KAN (MOI = 0.001, (**I**)) or with CFT073 (MOI = 0.001, (**J**)), as indicated. After 24 h of culture, we removed most of the bacteria by centrifugation and added gentamicin (400 µg/mL) for another 3 h to further reduce extracellular bacteria. We then monitored P2 promoter activity of cell-associated or non-associated bacteria by fluorescence microscopy ((**I**,**J**) left panels). White rectangles mark zoomed insets. Corresponding phase contrast pictures are also shown ((**I**,**J**), right panels).

We then investigated whether P2 activity increases further in bacteria with direct contact to THP-1 cells. We co-cultured CFT073 pPc2398:gfpmut2:KAN or CFT073 with THP-1 cells for 24 h and analyzed the fluorescence intensity of the reporter strain in the bacteria or THP-1 gate by flow cytometry (Figure 4A). The results demonstrated that the fluorescence intensity of THP-1 cells increased around 10-fold in the presence of CFT073 pPc2398:gfpmut2:KAN but not in the presence of CFT073 (Figure 4B–H). We repeated the experiment and centrifuged and washed the culture after around 24 h to enrich for eukaryotic cells and added gentamicin for another 3 h of culture. We observed by fluorescence microscopy that cell-associated CFT073 pPc2398:gfpmut2:KAN illuminated brightly in contrast to CFT073 pPc2398:gfpmut2:KAN not associated with THP-1 cells (Figure 4I) or the negative control CFT073 (Figure 4J). 

### 2.2. Potassium and Sodium Salts Induce the tcpC Promoter

**Figure 5:**
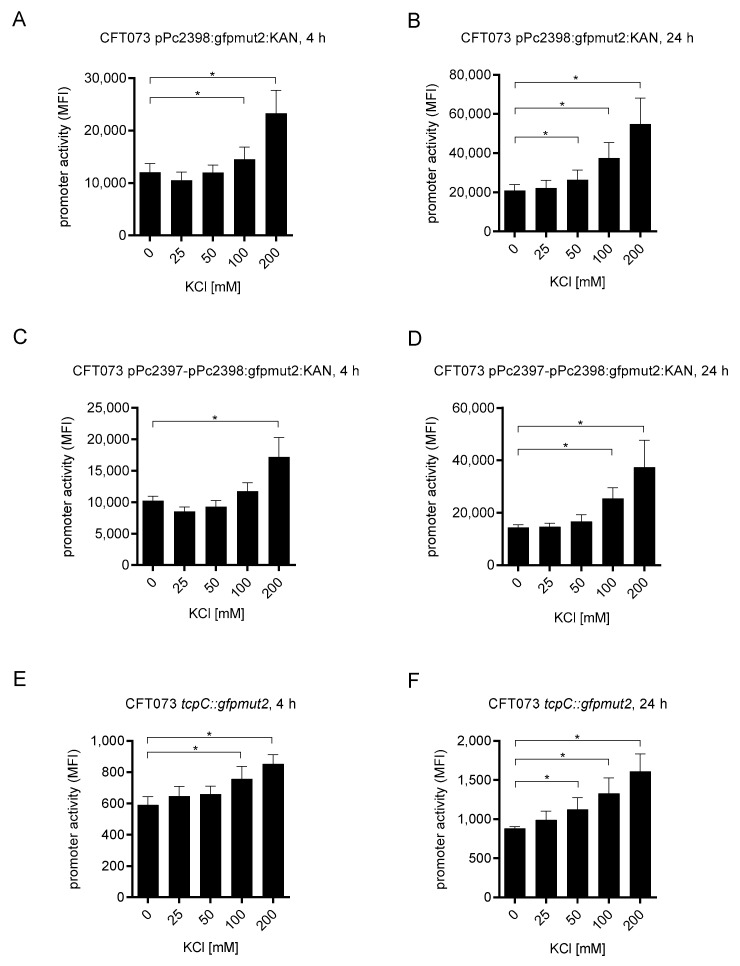
CFT073 senses potassium chloride. We cultured CFT073 pPc2398:gfpmut2:KAN (overnight culture diluted 1:6) in M9-minimal medium in the absence or presence of increasing amounts of potassium chloride for 4 (**A**) or 24 h (**B**) and determined the P2 promoter activity by flow cytometry. We repeated the experiment with CFT073 pPc2397-Pc2398:gfpmut2:KAN (overnight culture diluted 1:6) to test a possible influence of the P1 promoter on P2 (**C**,**D**) and with CFT073 *tcpC::gfpmut2* to test a chromosomal reporter strain (**E**,**F**). Graphs (**A**,**B**) depict 16 independent experiments, (**C**,**D**) depict four independent experiments, and (**E**,**F**) depict three independent experiments. Each experiment was performed with three replicates. * *p* < 0.05, ANOVA post-hoc Tukey.

Due to the strong cell-associated activity of P2 we explored the effects of potassium chloride on the activity of the promoter since potassium is the dominant intracellular cation. In addition, we analyzed the influence of sodium chloride on P2 induction since this salt generates the antibacterial hypersalinity of the kidneys [13]. Titration of potassium chloride within the physiological range of extracellular and intracellular concentrations revealed that the salt increased the promoter activity dose-dependently upon 4 but also 24 h of culture (Figure 5A,B and Figure S3). We repeated the experiment with the reporter construct pPc2397-Pc2398:gfpmut:KAN containing, in addition to the promoter P2, the promoter P1, which may generate a polycistronic mRNA encompassing *c2397* and *tcpC* (*c2398*). While we described earlier the transcription start of P2 in the 5′ UTR of the gene *c2397* [12], we now determined the transcription start of P1 using a 5′ RACE PCR approach and found two different transcription start sites, one 57 and the other one 13 bp before a putative start codon of *c2397* (Figure S4). Increasing potassium chloride concentration also stimulated the pPc2397-Pc2398:gfpmut:KAN (P1 + P2) reporter construct (Figure 5C,D) with no real difference to pPc2398:gfpmut:KAN (P2). Thus, the presence of P1 did not influence the potassium-driven activity of P2. Moreover, potassium chloride did not stimulate the P1 promoter in a preliminary experiment (data not shown). We also tested the chromosomal reporter strain CFT073 *tcpC::gfpmut2* which, importantly, responded significantly and dose-dependently to increasing potassium chloride concentrations upon 4 and 24 h of culture (Figure 5E,F).

**Figure 6:**
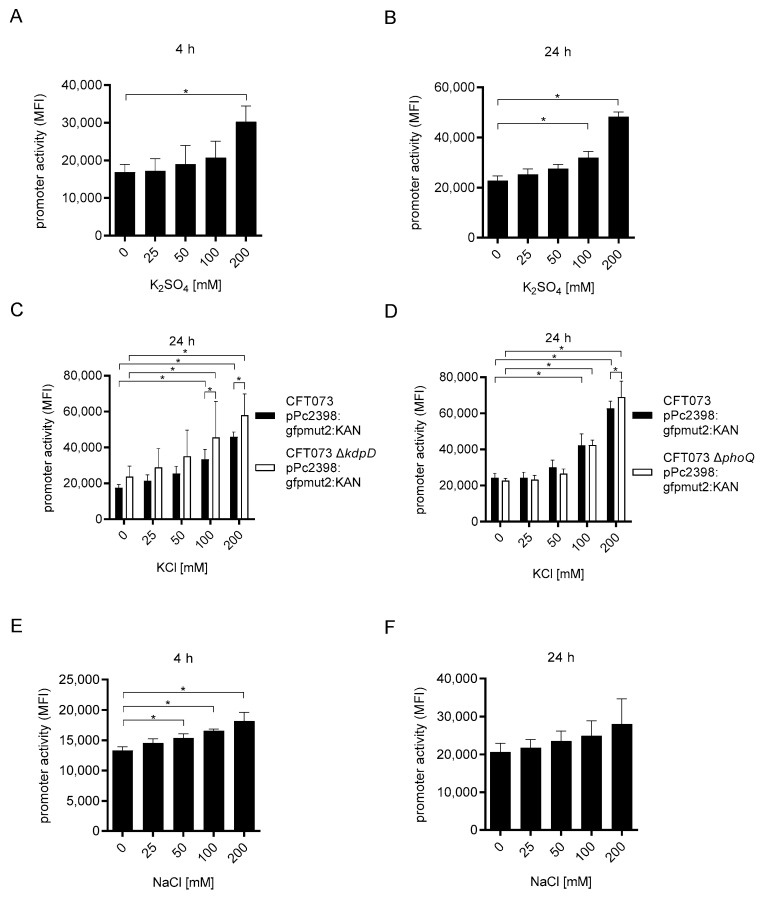
Potassium and sodium induce P2. We cultured CFT073 pPc2398:gfpmut:KAN (overnight culture diluted 1:6) in M9-minimal medium in the absence or presence of increasing amounts of potassium sulfate as indicated for 4 (**A**) or 24 h (**B**). To test a possible involvement of the two-component systems KdpD/E (**C**) and PhoQ/P (**D**) in potassium detection, we cultured the corresponding gene-deficient strains CFT073 Δ*kdpD* pPc2398:gfpmut:KAN and CFT073 Δ*phoQ* pPc2398:gfpmut:KAN in the absence or presence of increasing amounts of potassium chloride as indicated and compared their response with the wild-type CFT073 pPc2397:gfpmut:KAN strain. Finally, we cultured CFT073 pPc2398:gfpmut:KAN in the absence or presence of increasing amounts of sodium chloride as indicated for 4 (**E**) or 24 h (**F**). In all experiments we determined P2 promoter activity by flow cytometry. Graphs (**A**,**B**,**D**) depict three, (**C**) four, (**E**) six, and (**F**) five independent experiments. Experiments in (**A**,**B**,**E**,**F**) were performed with one and, in (**C**,**D**), with three replicates. * *p* < 0.05, ANOVA post-hoc Tukey (**A**,**B**,**E**,**F**), two-way ANOVA and Sidak’s multiple comparison test (**C**,**D**).

We then replaced potassium chloride with potassium sulfate to explore whether the potassium or chloride ion is sensed by CFT073. We found that potassium sulfate induced P2 dose-dependently and significantly after 4 and 24 h of culture (Figure 6A,B) and comparably to potassium chloride (Figure 5A,B) indicating that the potassium ion induced the P2 promoter. 

We then tested whether the two-component system KdpD/E controlling the expression of the high-affinity potassium uptake system Kpd [18,19], but also PhoQ/P, described above, influence the induction of P2 by potassium chloride. Potassium chloride induced the P2 reporter plasmid dose-dependently and significantly in both corresponding knock-out strains CFT073 Δ*kdpD* (Figure S2B) and CFT073 Δ*phoQ* (Figure S2C) as in the wild-type CFT073 strain (Figure 6C,D). We observed a statistically significant difference for P2 induction between CFT073 Δ*kdpD* and wild-type CFT073 at 100 and 200, and CFT073 Δ*phoQ* and wild-type CFT073 at 200 mM potassium chloride, respectively. Possibly, both two-component systems participate in P2 induction to a minor degree. 

Finally, we replaced the potassium ion with the sodium ion and found that sodium chloride also induced the P2 promoter dose-dependently and significantly after 4 h of culture (Figure 6E), albeit more weakly than potassium chloride (Figure 6A). Sodium chloride also increased P2 activity after 24 h of culture, although the differences were no longer significant (Figure 6F).

Taken together, CFT073 appears to sense potassium and sodium ions, which subsequently leads to the induction of the P2 promoter. 

### 2.3. Bacterial Density Induces the tcpC Promoter

**Figure 7:**
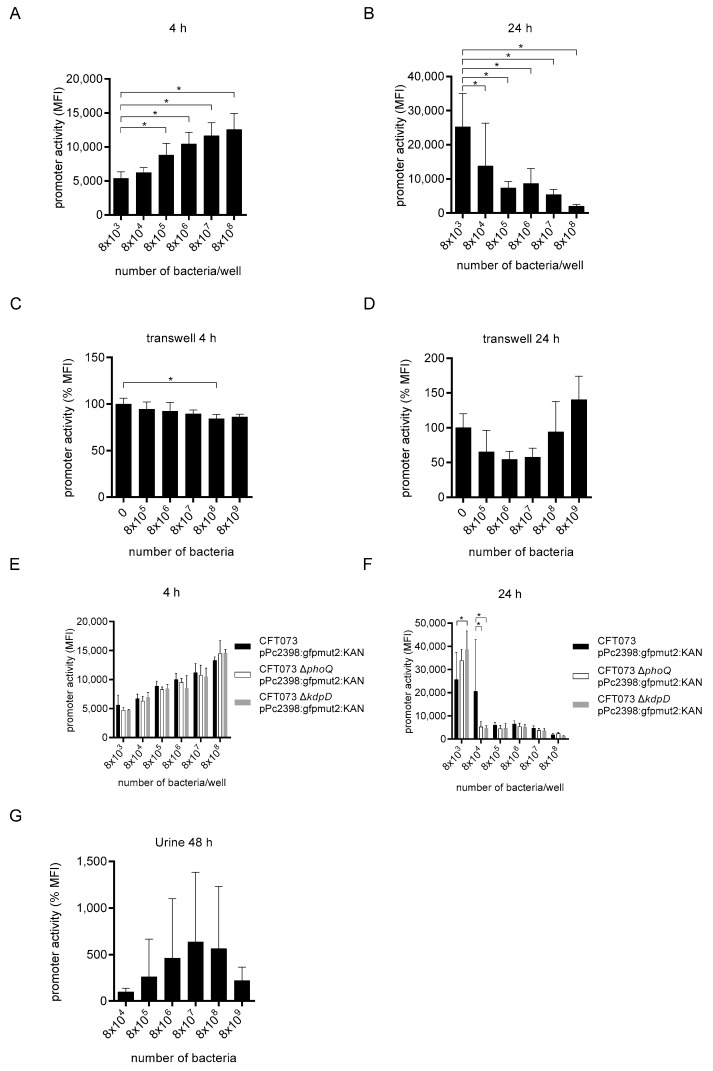
The bacterial density activates P2. We cultured CFT073 pPc2398:gfpmut:KAN in McCoy medium in the indicated amounts for 4 (**A**) or 24 h (**B**). We repeated the experiment using transwell cultures with CFT073 pPc2398:gfpmut:KAN (3.2 × 10^7^ bacteria/well) in the absence or presence of increasing amounts of CFT073 as indicated for 4 (**C**) and 24 h (**D**). We analyzed the influence of the two-component systems KdpD/E and PhoQ/P to sense bacterial density by repeating the experiment in A and B with the strains CFT073 pPc2398:gfpmut:KAN, CFT073 Δ*kdpD* pPc2398:gfpmut:KAN or CFT073 Δ*phoQ* pPc2398:gfpmut:KAN for 4 (**E**) or 24 h (**F**). In (**G**) we cultured CFT073 pPc2398:gfpmut:KAN in the indicated amounts for 48 h in urine. In all experiments we determined the P2 promoter activity by flow cytometry. Graphs (**A**,**B**) depict nine independent experiments, each with one replicate, (**C**) depicts two independent experiments, each with two replicates, (**D**) depicts three independent experiments with two replicates, and (**E**–**G**) depict three independent experiments. Experiments in (**E**,**F**) were performed with one, in (**G**) with four replicates. The measured promoter activity in (**C**,**D**) was normalized to the “0” control. * *p* < 0.05, ANOVA post-hoc Tukey (**A**–**D**,**G**), two-way ANOVA and Dunnett’s multiple comparison test (**E**,**F**).

During urinary-tract infections ascending bacterial pathogens multiply by several orders of magnitude. We therefore explored whether the bacterial density is also sensed by CFT073 with the consequence of P2 induction. Cultivation of the reporter strain CFT073 pPc2398:gfpmut2:KAN in increasing amounts induced the P2 promoter dose-dependently and significantly after 4 h of culture (Figure 7A and Figure S5A). We observed the converse effect after 24 h of culture, where the increasing bacterial density reduced the promoter activity dose-dependently and significantly (Figure 7B and Figure S5B). Thus, the influence of the bacterial density on the P2 promoter activity was time-dependent, an early induction vs. a late repression. Transwell cultures with titrated CFT073 numbers and constant amounts of the reporter strain CFT073 pPc2398:gfpmut2:KAN did not result in induction of the P2 promoter after 4 h of culture (Figure 7C) and reduced the promoter activity after 24 h of culture at a CFT073 density of 8 × 10^6^ bacteria/well to some extent but not significantly (Figure 7D). Thus, the bacterial density influenced the P2 promoter activity in a contact-dependent manner. Both two-component systems KdpD/E and PhoQ/P did not influence the induction of the P2 promoter by the bacterial density (Figure 7E) and had only a minor effect on its repression (Figure 7F). We then tested the influence of the bacterial density on the P2 promoter activity in human urine. We found that increasing bacterial numbers up to 8 × 10^6^ bacteria induced the promoter activity, however the differences were not significant (Figure 7G). Taken together, the P2 promoter activity is clearly regulated by the bacterial density in a time- and contact-dependent manner. 

### 2.4. Identification of P2 Promoter Regulatory Proteins

**Figure 8:**
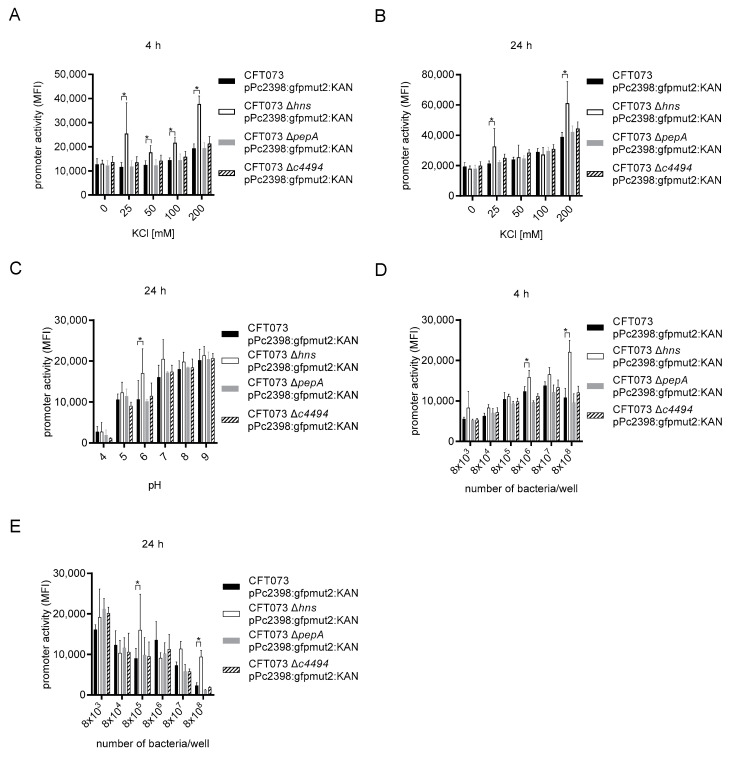
H-NS acts as negative regulator of P2. We cultured CFT073 pPc2398:gfpmut:KAN (overnight culture diluted 1:6), CFT073 Δ*hns* pPc2398:gfpmut:KAN (overnight culture diluted 1:6), CFT073 Δ*pepA* pPc2398:gfpmut:KAN (overnight culture diluted 1:6), or CFT073 Δ*c4494* pPc2398:gfpmut:KAN (overnight culture diluted 1:6) in M9 minimal medium in the absence or presence of increasing amounts of potassium chloride for 4 (**A**) or 24 h (**B**), or increasing pH values for 24 h (**C**), or in McCoy medium in the indicated amounts for 4 (**D**) or 24 h (**E**). In all experiments we determined the P2 promoter activity by flow cytometry. Graphs (**A**,**B**) depict six independent experiments, (**C**–**E**) depict three independent experiments. Each experiment was performed with one replicate. * *p* < 0.05, ANOVA post-hoc Tukey.

To identify proteins regulating the P2 promoter, we amplified the 240 bp DNA element starting at position 2202535 and ending at position 2202775 of the chromosome of CFT073 just 5′ of the *tcpC* gene (*c2398*) by PCR. A DNA fragment from the *lacZ* promoter of equal size served as control. We then precipitated DNA-binding proteins from a CFT073 lysate and identified those, which only bound to the P2 but not to the *lacZ* promoter in at least two independent experiments by mass spectrometry. This approach resulted in the identification of several proteins listed in Table 1. Three DNA-binding proteins with the highest number of unique peptides identified by mass spectrometry, i.e., cytosol aminopeptidase (PepA), DNA-binding protein H-NS, and putative transcriptional regulator (c4494), were chosen to generate the corresponding gene-deficient CFT073 strains CFT073 Δ*pepA* (Figure S2C), CFT073 Δ*hns* (Figure S2C), and CFT073 Δ*c4494* (Figure S2D). Comparison of these gene-deficient strains with wild-type CFT073 revealed that the CFT073 Δ*hns* strain responded significantly more strongly to potassium chloride after 4 h of stimulation while the response of the other two gene-deficient strains did not differ from the wild-type strain (Figure 8A). The phenotype of the CFT073 Δ*hns* strain was weaker after 24 h of culture (Figure 8B). As we reported earlier, high pH values stimulated the P2 promoter [12]. We therefore compared the P2 promoter activity of the three different gene-deficient P2 reporter strains upon stimulation with increasing pH. Again, CFT073 Δ*hns* responded significantly more strongly at pH6 compared to all other strains (Figure 8C). We then titrated the bacterial density of the three different gene-deficient P2 reporter strains and found again that CFT073 Δ*hns* responded significantly stronger after 4 h of culture than all other strains (Figure 8D). Moreover, the reduction of the P2-reporter activity after 24 h of culture at the highest density was significantly lower compared to all other strains (Figure 8E). Taken together, these data provide evidence that H-NS dampens the activity of the P2 promoter. 

## 3. Discussion

We show here that the uropathogenic *E. coli* strain CFT073 was able to sense uro-epithelial T24/83 and monocytic THP-1 cells leading to the induction of the *tcpC* promoter P2. PMA-matured THP-1 cells induced the P2 promoter more efficiently as less time was required to provoke this response. CFT073 cells, which were associated to THP-1 cells, responded the most. While the stimulation of P2 by eukaryotic cells with the exception of matured THP-1 cells required 24 h of culture, potassium chloride induced P2 after only 4 h of incubation. CFT073 sensed the potassium and not the chloride ion since potassium sulfate stimulated P2 equally. In addition to potassium chloride sodium chloride also induced P2, although somewhat weaker. Furthermore, CFT073 sensed the bacterial density by an early induction and later repression phase of the P2 promoter. Finally, we defined H-NS as a negative regulator of P2 upon induction by different stimuli.

We based our selection of stimuli for P2 on their presence during a urinary tract infection [13,20,21]. P2 controls the transcription of the *tcpC* gene (*c2398*), whose corresponding protein impairs two important pattern-recognition receptors and their signaling chains, i.e., TLR4/MyD88 and NLRP3/caspase-1 [4,5]. To reduce innate immune responses in time, we reasoned that cell types such as epithelial cells and monocytes, participating in local defense, should trigger P2 [22,23,24,25]. The mechanisms involved in host cell recognition by CFT073 are, so far, ill-defined, but appear to differ. Thus, the response of CFT073 induced by the uroepithelial cell line T24/83 requires cell contact, while P2 induction by the monocytic cell line THP-1 is contact-independent, presumably involving a soluble factor. It is also unclear which sensor leads to the activation of the P2 promoter on the pathogen’s site. We reasoned that the two-component system PhoQ/P, which recognizes macrophages and antibacterial peptides [14,17], might be involved. However, the CFT073 Δ*phoQ* strain responded like the wild-type strain. The strongest induction of P2 occurred in CFT073 in close association with THP-1 cells, i.e., either adherent to or intracellular in THP-1 cells. P2 induction was substantially stronger when compared to non-associated bacteria. Taken together, a relationship might exist between the cellular danger imposed on CFT073 and P2 induction—the greater the danger for CFT073, the greater the promoter induction. Accordingly, uroepithelial T24/83 cells exert a low level of danger and this level increases from monocytic THP-1 cells to PMA-matured THP-1. The highest danger level is exerted in close association with THP-1 cells.

The maximal P2 induction in association with THP-1 cells guided us to analyze potassium for its ability to induce P2 as it represents the dominant intracellular ion, is released during inflammasome activation, and CFT073 possesses the two-component system KdpD/E, which controls expression of the high-affinity potassium uptake system Kdp [19,26,27]. A concentration of 50 to 200 mM of potassium chloride significantly induced P2. By exchanging potassium chloride with potassium sulfate we demonstrated that the potassium ion, and not the chloride ion, stimulates P2. We reasoned that the two-component system KdpD/E [18,19] would influence P2 induction and found that the CFT073 Δ*kdpD* strain responded significantly stronger than the wild-type strain in cases of high potassium chloride concentrations. However, we detected no fundamental difference between the two strains in their ability to induce P2 upon stimulation with potassium chloride. Possibly, the constitutively expressed potassium-uptake systems, Trk and Kup, might be more relevant for P2 induction by potassium [19,28]. 

Our previous work demonstrated that, like P2, the 5′ untranslated region of *c2397*, which we designated as promoter P1, responded to high pH values and glucose concentrations of 60 mmol/L and was deactivated by FeSO_4_ [12]. The response was, in each case, considerably weaker compared to P2. To confirm P1 as promoter we determined its transcription start site in this study. While this approach verified P1 as promoter, our efforts to demonstrate a polycistronic transcript starting at P1 and including the genes 2397 and *tcpC* (*c2398*) were so far not convincing (data not shown). In contrast to pH and glucose, a preliminary experiment indicated that potassium did not significantly induce the P1 promoter (data not shown) and P1 did not influence P2 as shown here. Thus, further research is required to explore whether P1 participates in the transcription of the *tcpC* gene. Importantly, potassium chloride also activated the chromosomal reporter strain CFT073 *tcpC::gfpmut2*, demonstrating that its promoter-inducing ability was not due to an artefact caused by a multi-copy plasmid. 

The kidney reabsorbs the vast majority of filtrated sodium and by that creates a high interstitial concentration of sodium [29,30]. Interestingly, the renal sodium gradient appears to generate an antibacterial shield [13]. Moreover, high sodium levels strengthen immune responses although immunosuppressive findings were also reported [31,32]. These findings prompted us to evaluate whether sodium would induce the P2 promoter. We found that sodium chloride concentrations between 50 and 200 mM stimulated P2 significantly. These and higher concentrations exist in the kidney and are possibly sensed by CFT073 to induce TcpC. Subsequently CFT073 causes kidney abscesses in a TcpC-dependent manner in vivo [5,10]. 

During a urinary tract infection, UPECs multiply to high numbers and patients’ urine typically contains at least 10^5^ bacteria per ml. Moreover, UPECs grow rapidly in pooled human urine [33]. We thus wondered whether P2 induction would depend on bacterial burden. We found a significant contact- and dose-dependent activation of P2 after 4 h of co-incubation. To our surprise, the opposite occurred after a culture period of 24 h—in this case the bacterial density induced a significant contact- and dose-dependent suppression of P2. The reason for this opposing behavior is unclear and requires further investigation. *E. coli* communicates via the autoinducer 2 (AI-2) in a density-dependent manner [34]. The gene responsible for AI-2 production was identified as *luxS* [35] and is also present in the CFT073 genome (c3243). AI-2 is secreted and controls the expression of virulence factors such as type-3 secretion systems in enterohaemorrhagic and enteropathogenic *E. coli* [36]. Since P2 induction requires cell contact, AI-2 is presumably not involved in the cell-density-dependent regulation of P2.

Whether host cells, bacteria, potassium, and sodium use completely different or, at least in part, common mechanisms to stimulate P2 is at present unknown and requires further investigation. We hypothesize that the mechanisms differ since the nature of these stimuli is very diverse. 

To define transcription factors, which control the activity of the P2 promoter, we performed chromatin-precipitation experiments using a 240 bp DNA segment containing P2. So far only H-NS but not PepA or c4494 influenced the activity of the P2 promoter, as a variety of stimuli induced P2 significantly stronger in CFT073 Δ*hns*, while the CFT073 Δ*pepA* and CFT073 Δ*c4494* strains responded like the wild-type strain. This negative role of H-NS is in agreement with other reports demonstrating that H-NS acts as global transcriptional silencer of foreign DNA [37,38]. The manifold regulatory influence of H-NS on gene expression results from its interaction with curved DNA independently from a consensus sequence for DNA-binding [39,40]. Interestingly, we also precipitated StpA from the CFT073 lysate with the P2 promoter. This protein interacts with H-NS to form heterodimers, which bind DNA [37]. As the *tcpC* gene (*c2398*) is located within a *serU* island, which was horizontally transferred as foreign DNA into CFT073 and co-segregates with the high pathogenicity island of CFT073 [11], the suppressive influence of H-NS on the P2 promoter fits with its previously reported suppression of foreign DNA [37]. Mass spectrometry identified H-NS encoded by the gene c1701 of CFT073 genome but not by the *hns* gene (c2411) located within the *serU* island. Whether the latter protein is also involved in the suppression of genes of the *serU* island remains to be determined.

In summary this report demonstrates that the P2 promoter, regulating the immunosuppressive gene *tcpC*, is induced by the uroepithelial cell line T24/83, the monocytic cell line THP-1, the bacterial density, and the two abundant ions sodium and potassium and is negatively regulated by H-NS. We assume that the pathogen senses these environmental factors to orchestrate its pathogenicity. 

## 4. Materials and Methods

### 4.1. Bacterial Strains 

**Figure 9:**
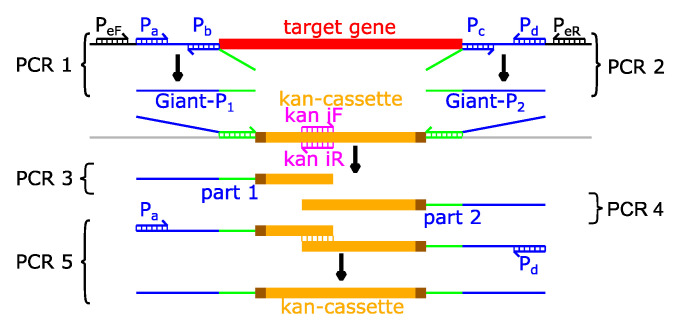
Strategy to generate gene-targeting cassettes. We used the primer pairs P_a_, P_b_ (PCR 1) and P_c_, P_d_ (PCR 2) to generate the “Giant” primers P_1_ and P_2_ which are homologous to the flanking regions of the target gene but also hybridize to the kanamycin cassette of pKD4. Using the primers P_1_ and kan iR (PCR 3) or P_2_ and kan iF (PCR 4) we amplified the 5′ and 3′ part of the kan-cassette. We then used the primers P_a_ and P_d_ (PCR 5) to fuse both parts and by that generating the complete gene targeting cassette. Primers P_ef_ and P_eR_ were used to verify the gene deficient mutant strains.

The UPEC strain CFT073 was purchased from ATCC (Manassas, VA, USA) [1]. The *tcpC*-deficient reporter strain CFT073 *tcpC::gfpmut2* was constructed using the λ-red system [41] as described in our previous study [12]. We used this system to generate the gene-deficient CFT073 strains CFT073 Δ*kdpD*, CFT073 Δ*phoQ*, CFT073 Δ*hns*, CFT073 Δ*pepA,* and CFT073 Δ*c4494* (Table 2, Figure S2). We amplified about 250 bp of DNA upstream and downstream of the target gene in two PCRs using two sets of primers P_a_/P_b_ and P_c_/P_d_ in each case (Figure 9, Table 3). Primers P_b_ and P_c_ both contain an overhang that is complementary to the flanking regions of the kanamycin (kan)-cassette located on the plasmid pKD4 (Table 4). The two PCR-products can then be used as “Giant-primers” P_1_ and P_2_ to amplify the kanamycin cassette of pKD4. Accordingly, we used P_1_ together with kan iR and P_2_ together with kan iF to amplify the 5′ part and the 3′ part of the kan-cassette, respectively. We then fused the two parts using the outer primers P_a_ and P_d_ (Figure 9). In the case of *kdpD* we did not produce the “Giant-primers” by PCR but ordered them as kdpD GP_1_ and kdpD GP_2_ (Table 3). The final PCR-product consisted of the kan-cassette flanked by two FRT-sequences and two long arms that were complementary to the flanking regions of the target gene. Next, we transformed electro-competent CFT073 with pKD46 carrying the λ red system under the control of an arabinose-inducible promoter and an ampicillin-resistance gene. Subsequently, we incubated the bacteria at 30 °C, to protect the heat-sensitive plasmid and selected positive clones on LB-agar plates containing ampicillin (amp). We then grew these bacteria in medium containing 1 mM arabinose and transformed them with the previously amplified gene targeting kan-cassette. We selected transformed clones on LB-agar plates containing kanamycin and verified the replacement of the target gene with the kan-cassette in a colony-PCR. To remove the kan-cassette from the genome, we transformed the clones with the plasmid pCP20 containing a FLP-recombinase. We selected positive clones with chloramphenicol (Cm) and verified them with colony PCR using the primers P_a_ and P_d_ or the external primers P_eF_ and P_eR_ (Figure S2). In a last step, the bacteria were cultured at 42 °C on LB-agar without antibiotics to remove all heat-sensitive plasmids. Clones that did not grow on LB-agar plates containing kan, amp, or cm were plasmid free and we used them to make glycerol stocks of the finished knockout mutants. 

### 4.2. Primers/Plasmids

All primers of this study are listed in Table 3 and came from Sigma-Aldrich (St. Louis, MO, USA). 

All plasmids used in this study are listed in Table 4. We described the generation of reporter plasmids previously [12].

### 4.3. Cell Lines

We used the human uroepithelial cell line T24/83 kindly donated by the Clinic for Urology (University Medical Centre Mannheim, Mannheim, Germany) and the human monocytic cell line THP-1 (CLS Cell Lines Service GmbH, Eppelheim, Germany).

### 4.4. Culture Media and Reagents

Luria-Bertani broth (LB) and LB agar were purchased from Roth (Karlsruhe, Germany). The 5×M9-minimal salt contained di-sodium hydrogen phosphate (33.9 g/L; Roth, Karlsruhe, Germany), potassium-dihydrogen phosphate (15 g/L; Merck, Darmstadt, Germany), sodium chloride (2.5 g/L; Merck, Darmstadt, Germany) and ammonium-chloride (5 g/L; Merck, Darmstadt, Germany). This M9-salt was diluted 1:5 and supplemented with magnesium sulfate (1 mM, Merck, Darmstadt, Germany), calcium chloride (0.1 mM; Merck, Darmstadt, Germany), glucose (0.4%, Merck, Darmstadt, Germany), thiamin-hydrochloride (10 µg/mL, Roth, Karlsruhe, Germany) and nicotinic acid (0.0025%, Roth, Karlsruhe, Germany). We bought McCoy and RPMI-1640 from Merck (Darmstadt, Germany) and FBS from Thermo Fisher Scientific (Waltham, MA, USA). Human urine was donated by healthy individuals. The urine was filtered (0.2 µm pore size) and used at the day of donation. Potassium chloride came from Merck (Darmstadt, Germany), potassium sulfate from Roth (Karlsruhe, Germany). Phorbol 12-myristate 13-acetat was bought from PromoCell (Heidelberg, Germany). We purchased *E. coli* LPS K235 from Merck (Darmstadt, Germany), kanamycin, ampicillin and gentamycin from AppliChem (Darmstadt, Germany) and chloramphenicol from Merck (Darmstadt, Germany). 

### 4.5. Culture of Bacteria

The *tcpC*-deficient reporter strain CFT073 *tcpC::gfpmut2*, and plasmid-transformed CFT073 reporter strains were cultured overnight at 37 °C and 200 rpm in LB medium or M9 minimal medium containing glucose in the presence of ampicillin (100 μg/mL) or kanamycin (25 μg/mL); wild-type CFT073 was cultured in the absence of antibiotics.

Bacterial growth was determined by measuring the optical density using an OD_600_ DiluPhotometer (IMPLEN, Munich, Germany).

### 4.6. Generation of Reporter CFT073 Strains

Then, 100 μL of electro-competent CFT073, CFT073 Δ*kdpD*, CFT073 Δ*phoQ*, CFT073 Δ*hns*, CFT073 Δ*pepA* or CFT073 Δ*c4494,* and 0.1–1.0 μL of plasmid-minipreps of pPc2397:gfpmut2:KAN, pPc2398:gfpmut2:KAN or p(Pc2397-Pc2398):gfpmut2:KAN were mixed and the plasmids electroporated (5 ms, 1700 V, Multiporator, Eppendorf, Germany) into the different CFT073 strains. Subsequently, 100 μL LB medium was added and the bacterial suspension was cultured on LB agar plates supplemented with kanamycin or ampicillin. We picked single colonies after overnight incubation.

### 4.7. Promoter-Stimulation Assays

We used the plasmid-based reporter strains CFT073 pPc2398:gfpmut2:KAN (P2 promoter), CFT073 Δ*kdpD* pPc2398:gfpmut2:KAN, CFT073 Δ*phoQ* pPc2398:gfpmut2:KAN, CFT073 Δ*hns* pPc2398:gfpmut2:KAN, CFT073 Δ*pepA* pPc2398:gfpmut2:KAN or CFT073 Δ*c4494* pPc2398:gfpmut2:KAN, and in case of stimulation with potassium chloride, additionally the plasmid-based reporter-strain CFT073 p(Pc2397-Pc2398):gfpmut2:KAN (P1 + P2 promoter) as well as the chromosomal reporter-strain CFT073 *tcpC::gfpmut2* to explore their promoter activity. 

We induced the promoter by co-culture or culture in transwells of CFT073 pPc2398:gfpmut2:KAN with titrated amounts of T24/83 or THP-1 cells as indicated in the figure legends. The latter cells were also matured with phorbol 12-myristate 13-acetate (100 ng/mL) for 3 days. The medium used for T24/83 or THP-1 cells was McCoy or RPMI-1640, respectively, supplemented with 10% FBS.

We also stimulated the promoter activity with titrated amounts of potassium chloride, potassium sulfate, or sodium chloride as indicated in the figure legends. We used the M9-minimal medium in these assays. 

Further, we explored the influence of the CFT073 density on the activity of the P2 promoter. We either titrated the number of the different reporter strains in McCoy medium or we used transwells with a constant number of the reporter strains and titrated amounts of CFT073.

Finally, we determined the promoter activity at increasing pH values by adjusting the pH of the used M9 salt with HCl or NaOH to the desired pH values. 

We determined in all cases the promoter activity after 4 and 24 h of culture by flow cytometry.

### 4.8. Analysis of the Promoter Activity by Flow Cytometry

To measure the activity of the P2 and P1 + P2 promoters, we pelleted 100 μL–1 mL of the samples by centrifugation (16,100× *g*, 2 min), removed the supernatant and resuspended the pellets in 200 μL 1 % PFA. After 30 min incubation, we replaced PFA with DPBS (400 µL) by centrifugation (16,100× *g*, 2 min), washed them once in DPBS, and stored the bacteria at 4 °C until analysis. We used the flow cytometer CytoFlex 3L PL (Beckman Coulter, Krefeld, Germany) to explore the fluorescence activity of the different CFT073 reporter strains. We analyzed the data with FlowJo (Ashland, OR, USA).

### 4.9. DNA-Precipitation of P2 Binding Proteins

We generated a biotinylated 240 bp DNA fragment of P2 and the lacZ promoter using PCR and the primer pairs tcpC 5′UTR_Biotin_fw, tcpC 5′UTR_rev and lacZ 5′UTR_Biotin_fw, lacZ 5’UTR_rev, respectively (Table 3). The lacZ promoter DNA fragment served as negative control. PCR amplicons were gel- purified, ethanol (70%)-precipitated and centrifuged (4 °C, 12,000× *g*, 5 min.). DNA-pellets were dried at room temperature and dissolved in double-distilled H_2_O. We coupled the purified DNA fragments to streptavidin-coated magnetic beads (Thermo Scientific, Dreieich, Germany). Before use, the beads were washed with BS/THES buffer (22 mM Tris HCl pH 7.5, 4.4 mM EDTA, 8.9% sucrose (*w*/*v*), 62 mM NaCl, 10 mM HEPES, 5 mM CaCl_2,_ 50 mM KCl, and 12% glycerol) in the presence of salmon sperm DNA (10 µg/mL) to impair unspecific DNA-binding. 

A 100 mL culture of CFT073 in M9-glucose-thiamine-NS, pH7 was pelleted, resuspended in 5 mL BS/THES buffer containing three tablets of complete mini protease inhibitor (Roche, Mannheim, Germany), lysed by five freeze–thaw cycles in liquid nitrogen, and subsequently sonicated five times. We incubated the bacterial lysate together with the DNA-coupled magnetic beads for 1 h in a vertical rotator. Beads were then pelleted (4000× *g*, 10 min), the supernatant discarded, washed twice in BS/THES and analyzed by mass spectrometry. 

### 4.10. Mass Spectrometry: In-Gel Digestion of Samples 

The protein samples were heated to 95 °C for 5 min and cooled on ice prior to loading onto NuPAGE 4–12% Bis-Tris Gels (Thermo Fisher Scientific; Waltham, MA, USA). SDS polyacrylamide gelelectrophoresis (SDS-PAGE) was performed according to the manufacturer’s specification. Proteins were fixed within the polyacrylamide matrix by incubating the entire gel in 5% acetic acid in 1:1 (vol/vol) water:methanol for 30 min. After Coomassie staining (60 min) the gel slab was rinsed with water (60 min) and each lane was excised and cut into small pieces.

Subsequently the proteins were in-gel destained (100 mM ammonium bicarbonate/acetonitrile 1:1 (vol/vol)), reduced (10 mM DTT), alkylated (50 mM iodoacetamide), and finally trypsin-digested by overnight incubation at 37 °C. The generated peptides were collected from the gel pieces, which were further subjected to a peptide extraction step with an acidic (1.5% formic acid) acetonitrile (66%) solution. Both peptide-containing samples were combined and dried down in a vacuum centrifuge.

### 4.11. Mass Spectrometry: Analysis 

Dried peptides were re-dissolved in 0.1% trifluoroacetic acid and loaded on a C18 precolumn (Acclaim; Thermo Fisher Scientific; Waltham, MA, USA) using a RSLCnano HPLC system (Thermo Fisher Scientific; Waltham, MA). Peptides were then eluted with an aqueous–organic gradient, resolved on a C18 column (Acclaim; Thermo Fisher Scientific; Waltham, MA, USA) with a flow rate of 300 nL/min, and electrosprayed into a LTQ Orbitrap XL mass spectrometer (Thermo Fisher Scientific; Waltham, MA, USA). A Triversa Automate (Advion, Ithaca, NY, USA) was used as ion source. Each scan cycle consisted of one FTMS full scan and up to seven ITMS-dependent MS/MS scans of the seven most intense ions. Dynamic exclusion (30 s), mass width (10 ppm), and monoisotopic precursor selection were enabled. All analyses were performed in positive ion mode. Extracted MS/MS spectra were searched against the Uniprot *E. coli* strain CFT073 database using the PEAKS search engine (Bioinformatics Solutions Inc., Waterloo, ON, Canada) accepting common variable modifications and one missed tryptic cleavage. Peptide tolerance was ±10 ppm and MS/MS tolerance was ±0.5 Da. All protein identification experiments were carried out using the corresponding decoy database and a false-discovery rate (FDR) of 1%.

### 4.12. Statistics

We used Prism 8.4.3 (GraphPad software, San Diego, CA, USA) to analyze experimental results for statistical significant differences. One-way ANOVA and Tukey Test as post-hoc was used to compare experimental groups. We used two-way ANOVA and Sidak’s multiple comparison test to compare responses between two bacterial strains. We considered *p* values of less than 0.05 as significant.

## Figures and Tables

**Table 1:** Identification of proteins binding to the P2 promoter.

Protein Name	Uniprot Entry	Score (−10 lgP)	Coverage (%)	Peptides	Unique Peptides	Avg. Mass
DNA-binding protein StpA	A0A0H2VCA0	211.11 138.62	40 24	5 2	5 2	15,360 15,360
C y t o s o l a m i n o p e p- t i d a s e	P68766	199.08 121.75	16 8	7 3	7 3	54,880 54,880
DNA-binding protein H -N S	P0ACF9	177.18 117.92	31 22	6 2	6 2	15,540 15,540
5-methyl-tetrahydropteroyltriglutamate-homocysteine methyltransferase	Q8FBM1	156.12 43.01	7 3	5 2	5 2	84,691 84,691
R4-like protein	Q8VR68	139.22 104.41	18 6	6 2	5 2	42,719 42,719
Uncharacterized protein	A0A0H2V7N7	139.20 75.95	12 7	3 2	3 2	34,655 34,655
P u t a t i v e t r a n s c r i p t i o n a l r e g u l a t o r (c4494)	A0A0H2VDP8	128.29 118.72 59.19	10 10 3	5 3 1	5 3 1	41,118 41,118 41,118
Succinate-CoA ligase (ADP-forming) subunit beta	P0A837	115.18 68.76	8 4	2 1	2 1	41,393 41,393
50S ribosomal protein L9	P0A7R2	109.27 44.09	25 15	3 2	3 2	15,769 15,769
Phospho-enolpyru-vate-protein phospho-transferase	A0A0H2VBQ5	84.47 69.18	3 2	1 1	1 1	55,207 55,207
IucD protein	A0A0H2VC61	77.64 34.30	7 3	2 1	2 1	50,897 50,897
30S ribosomal protein S10	P0A7R6	74.76 39.49	15 15	1 1	1 1	11,736 11,736
Branched-chain-amino-acid aminotransferase	P0AB81	69.80 60.31	7 7	2 2	2 2	34,094 34,094
Uncharacterized protein YheO	P64625	68.62 28.10	5 5	1 1	1 1	26,821 26,821
Transcriptional regulator MraZ	P65434	67.52 20.53	11 5	2 1	2 1	17,360 17,360
HTH-type transcriptional repressor CytR	P0ACN8	54.26 53.16	3 5	1 2	1 2	37,820 37,820
50S ribosomal protein L7/L12	P0A7K3	28.78 26.44	10 10	1 1	1 1	12,295 12,295

Number of result lines per protein represents the number of independent experiments. Listed proteins did not bind to the *lacZ* promoter, which was used as negative control. We selected the underlined genes to generate gene-deficient CFT073 strains.

**Table 2:** Bacterial strains.

Strains	Deleted Gene	Locus Tag	Function
CFT073	none		
CFT073 *tcpC::gfpmut2*	*tcpC*	c2398	Chromosomal *tcpC* reporter
CFT073 Δ*kdpD*	*kdpD*	c0780	Sensor protein KdpD, component of the potassium-sensing, two-component system KdpD/E
CFT073 Δ*phoQ*	*phoQ*	c1508	Sensor protein PhoQ, component of the macrophage-sensing, two-component system PhoQ/P
CFT073 Δ*hns*	*hns*	c1701	DNA-binding protein H-NS
CFT073 Δ*pepA*	*pepA*	c5360	Cytosol aminopeptidase
CFT073 Δ*c4494*	*c4494*	c4494	Putative transcriptional regulator

**Table 3:** Primer names and sequences. Upper case letters represent overhangs.

Designation	Sequence
phoQ a	5′-catttaaccggaaaaaag-3′
phoQ b	5′-GCAGCTCCAGCCTACACttatcactacatcaaggc-3′
phoQ iR	aacgttacgacaaatatc
phoQ c	5′-GGAGGATATTCATATGtcagcgcaattcgaacag-3′
phoQ d	5′-gacgaagtgatcaaactg-3′
kdpD eF	gataatcatccgctccgg
kdpD GP1	CGCAGAAAGCGACGAATAGCCTGTTCATCTTC-AACAATCAGAACGTTTGTCACgtgtaggctggagctgc
kdpD iF	ccagcgttaaccactctt
kdpD iR	aagagtggttaacgctgg
kdpD GP2	GCCGGTCGTCAACATTGTCGAACTCAATCTG-GCACTGGATAAGCTTcatatgaatatcctccttagttcc
kdpD eR	taaacagatagccgcacg
hns a	5′-attatctccccataaaatg-3′
hns b	5′-GCAGCTCCAGCCTACACatctcaaacttatattggg-3′
hns c	5′-GGAGGATATTCATATGtcttttgtagattgcac-3′
hns d	5′-actgcggtaataaattag-3′
pepA eF	cccgcaccagatatcttatg
pepA a	5′-cgtaaaaactcgtcttttgc-3′
pepA b	5′-GCAGCTCCAGCCTACACgctcctgaatcttaaagac-3′
pepA c	5′-GGAGGATATTCATATGtcaggctgtgttgttattg-3′
pepA d	5′-catatatggggcttcttgtg-3′
pepA eR	gtccagaaggtagaacgttg
c4494 a	5′-ggtgtttgctgacagctata-3′
c4494 b	5′-GCAGCTCCAGCCTACACttctcatgggatggaatagc-3′
c4494 c	5′-GGAGGATATTCATATGaagggaaaataagcgtaatgttc-3′
c4494 d	5′-gccaggggttatcttctg-3′
kan iF	gaggctattcggctatgactg
kan iR	cagtcatagccgaatagcctc
GSP1	5′-tgagcatggggattcttact-3′
GSP2	5′-ccaagtcatgaaatgcgata-3′
GSP3	5′-tgcccgtacacgtattacag-3′
AAP (Abriched Anchor Primer)	5′-ggccacgcgtcgactagtacgggiigggiigggiig-3‘
AUAP (Abriched universal Amplification Primer)	5′-ggccacgcgtcgactagtac-3′
tcpC 5′UTR_Biotin_fw	5′-[Btn]gcaggagtctatggtaacg-3′
tcpC 5′UTR_rev	5′-catatgctatcacattttgag-3′
lacZ 5′UTR_Biotin_fw	5′-[Btn]agaaaaaccacccttccg-3′
lacZ 5’UTR_rev	5′-catagtcatagctgtatcctgtg-3′

**Table 4:** Plasmids.

Bacterial Host	Construct	Plasmid Back-Bone	Resistance	Plasmid Name	Function
CFT073	Pc2397:gfpmut2	pUA66	kan ^†^	pPc2397:gfpmut2:KAN	P1 reporter
CFT073	Pc2398:gfpmut2	pUA66	kan	pPc2398:gfpmut2:KAN	P2 reporter
CFT073	Pc2397-Pc2398:gfpmut2	pUA66	kan	p(Pc2397-Pc2398):gfpmut2:KAN	P1+P2 reporter
CFT073			kan, amp ^‡^	pKD4	Carries the kanamycin cassette flanked by FRT-sequences
CFT073			amp	pKD46	Carries the arabinose-inducible λ-red-system
CFT073			cm ^¶^, amp	pCP20	Carries the FLP-recombinase

^†^ kanamycin, ^‡^ ampicillin, ^¶^ chloramphenicol.

## Data Availability

Data available on request from the authors.

## Supplementary Materials

The following supporting information can be downloaded at: https://www.mdpi.com/article/10.3390/ijms23031148/s1.
