# Supplementary material for: The Promoter of the Immune-Modulating Gene TIR-Containing Protein C of the Uropathogenic Escherichia coli Strain CFT073 Reacts to the Pathogen’s Environment
Source: Int J Mol Sci. 2022 Jan 20;23(3):1148. doi: 10.3390/ijms23031148 (PMC8835471; doi:10.3390/ijms23031148)
Supplement: Supplementary file 1 [file ijms-23-01148-s001.zip › ijms-1543260-supp.pdf]

Supplementary Figures

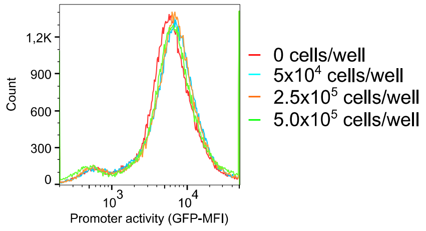

Figure S1. Flow cytometry analysis of P2 induction by matured THP-1 cells. The graph depicts representative flow cytometry histograms of the experiments described in Figure 2F.

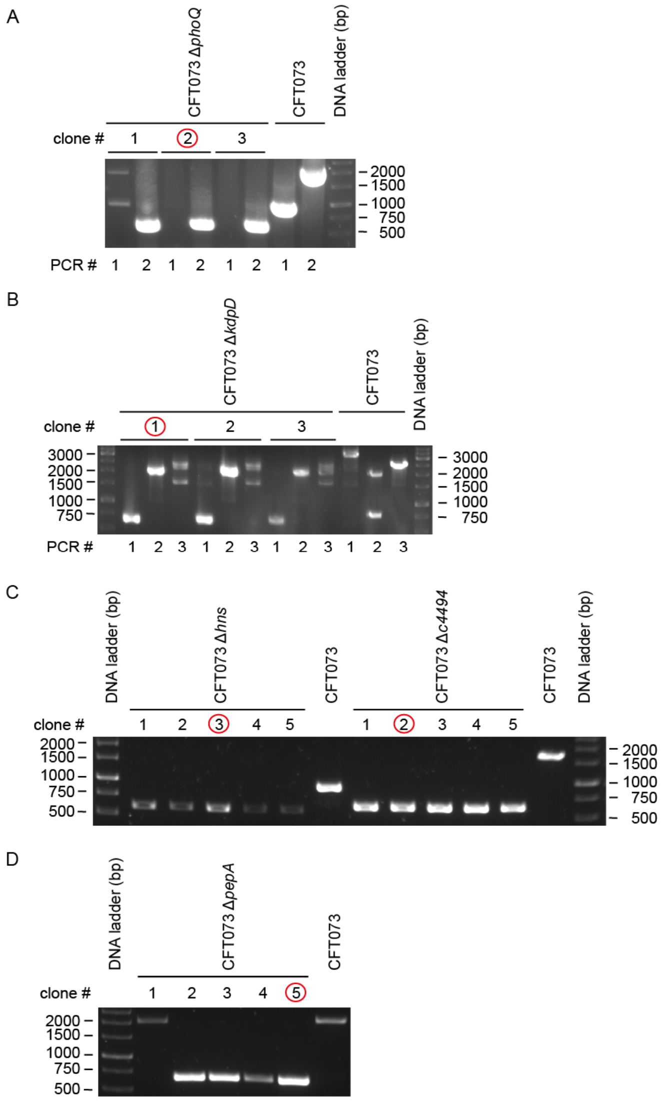

Figure S2. PCR-based verification of gene-deficient CFT073 mutants. We verified the successful gene deletion of CFT073 Δ*phoQ* (**A**), CFT073 Δ*kdpD* (**B**), CFT073 Δ*hns*, CFT073 Δ*c4494* (**C**), and CFT073 Δ*pepA* (**D**) by PCR. In (A) we used the primer pairs phoQ a/phoQ iR (PCR #1) and phoQ a/phoQ d (PCR #2), in (B) kdpD eF/kdpD eR (PCR #1), kdpD eF/kdpD iR (PCR #2), kdpD iF/kdpD eR (PCR #3), in (C) hns a/hns d and c4494 a/c4494 d, and in (D) pepA eF/pepA

eR. Sequences of all primers are listed in Table 3. Red circles in (A–D) represent the clone of each gene-deficient CFT073 mutant that we used in experiments.

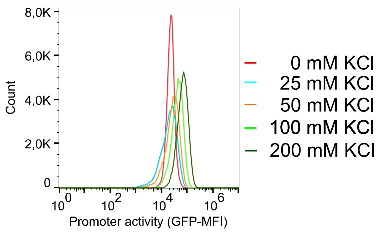

Figure S3. Flow-cytometry analysis of P2 induction by potassium chloride. The graph depicts representative flow-cytometry histograms of the experiments described in Figure 5B.

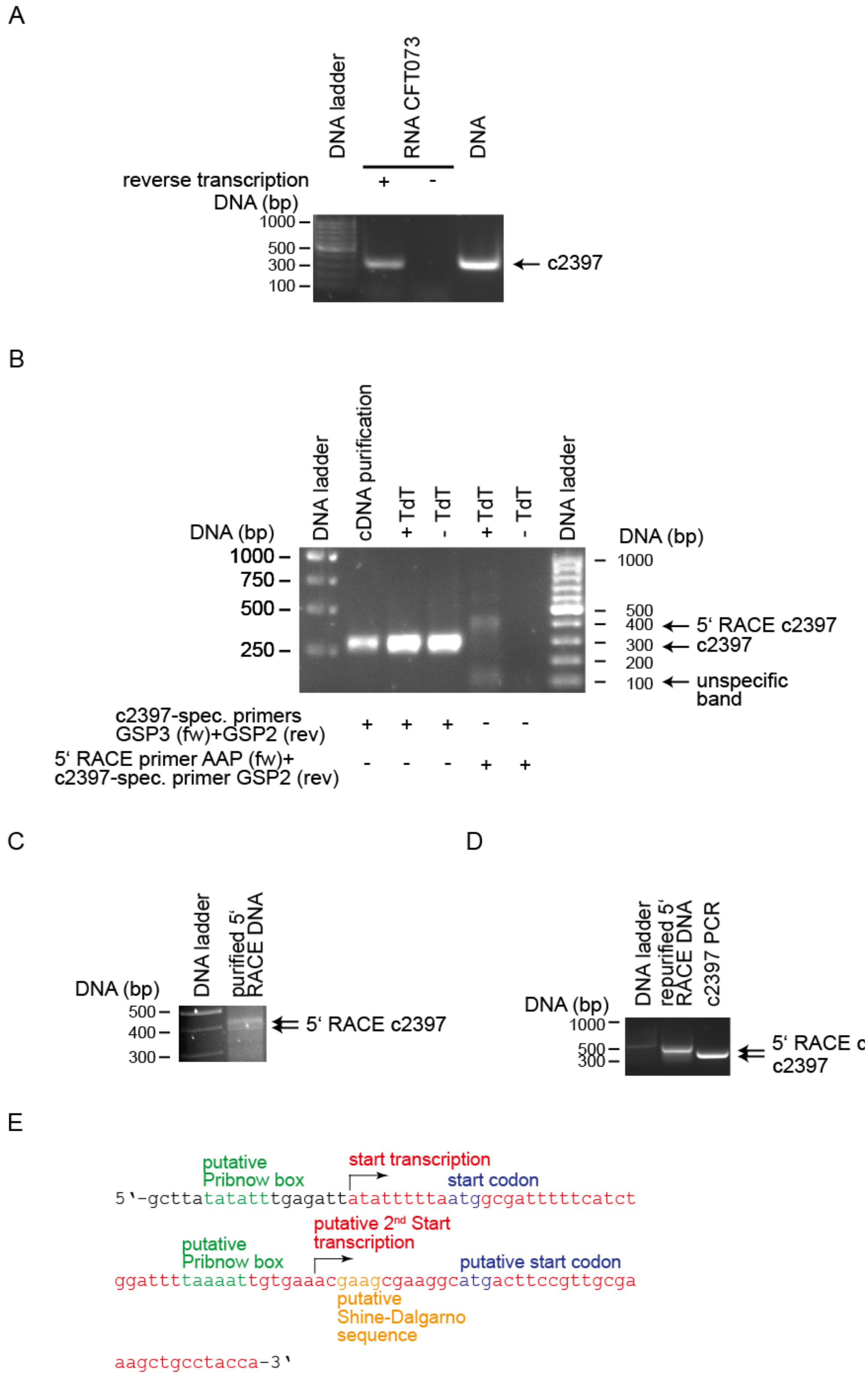

Figure S4. Detection of *c2397* transcripts and genomic position of the P1 promoter. We cultured CFT073 overnight in M9-minimal medium containing glucose (0.4% = 22.2 mmol/l), pH8 until an OD_{600} of about 0.65 was reached. (**A**) We prepared RNA and reverse-transcribed, or not, (as indicated) using the primer GSP1 and performed a PCR using the primers GSP3 (forward) and GSP2 (reverse) to amplify a fragment of the *c2397* mRNA. (**B**) For 5′ RACE PCR we used the 5′ RACE System for Rapid Amplification of cDNA Ends, Version

2.0 from Invitrogen. We synthesized cDNA using the primer GSP1. After tailing the cDNA with oligo C by terminal deoxynucleotidyl transferase (TdT), or not, we performed a control PCR using the primers GSP3 and GSP2 to detect *c2397* mRNA. To detect the start of the *c2397* transcript we used the forward primer AAP and the reverse primer GSP2. We detected a weak transcript only in case of addition of oligo C, as expected. We re-amplified the 5′ RACE *c2397* band depicted in (B) (lane 5) using the forward primer AUAP and GSP2 (reverse primer) as shown in (C). We re-amplified the product in (C) (lane 2) using the primers AUAP and GSP2 but also GSP3 and GSP2 as depicted in (D) (lane 2) and (D) (lane 3), respectively. (E) Sequencing of the PCR-product depicted in (D) (lane 2) revealed two transcript starts at position 2201194 and 2201238 of the CFT073 genome, or 57 and 13 bp before a putative start codon of *c2397*.

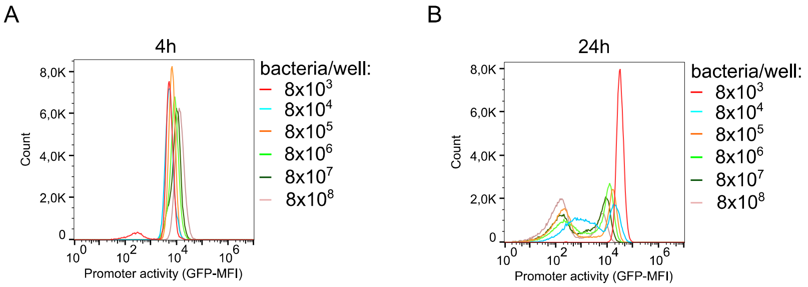

Figure S5. Flow cytometry analysis of P2 induction by the bacterial density. The graph depicts representative flow cytometry histograms of the experiments described in Figure 7A,B.
